# Mapping a shared genetic basis for neurodevelopmental disorders

**DOI:** 10.1186/s13073-017-0503-4

**Published:** 2017-12-14

**Authors:** Matthew Jensen, Santhosh Girirajan

**Affiliations:** 1 Bioinformatics and Genomics Graduate Program, The Huck Institutes of Life Sciences, Pennsylvania State University, University Park, PA 16802 USA; 2 Department of Biochemistry and Molecular Biology, Pennsylvania State University, University Park, PA 16802 USA; 3 Department of Anthropology, Pennsylvania State University, University Park, PA 16802 USA

**Keywords:** Causative variants, Complex disease, Copy number variants, Gene discovery, Modifiers, Neurodevelopmental disorders

## Abstract

Distinct neurodevelopmental disorders have a common genetic etiology that explains the high degree of comorbidity among these disorders. A recent study sought to identify copy number variants across five neurodevelopmental disorders, and detected an enrichment for chromosome 9p24.3 duplication encompassing *DOCK8* and *KANK1* in affected individuals. Such large-scale studies will help uncover additional causative and modifier loci within common pathways, which will enable the development of therapeutic targets for the treatment of multiple disorders.

See related research 10.1186/s13073-017-0494-1

## Genetic underpinnings supersede disease classifications

Neurodevelopmental disorders, such as autism, intellectual disability, schizophrenia, and epilepsy, are characterized by strong clinical comorbidity, which suggests a common genetic etiology across the diverse group of disorders. Recent studies have found that inheritance of one neurodevelopmental disorder also confers an increased risk for other disorders within the same family. For example, monozygotic twins were found to have a higher propensity to develop attention-deficit/hyperactivity disorder (ADHD) or a learning disability if their co-twin had autism [1], whereas individuals whose relatives had schizophrenia were more likely to develop bipolar disorder, depression, and autism compared to the general population [2]. Therefore, it is increasingly clear that the genetic underpinnings for these disorders conform to neither the nosological “models” nor the diagnostic criteria for disease classification according to the Diagnostic and Statistical Manual of Mental Disorders [1]. Major sources for the shared heritability of these disorders include single-nucleotide variants (SNVs) and copy number variants (CNVs), with rare recurrent CNVs having an especially large effect size for neurodevelopmental disorders. Many rare CNVs and SNVs have been implicated in different neurodevelopmental disorders, but examination of multiple disease cohorts in a single study allows for a more robust identification of the shared genetic basis of these disorders.

## The same needles in different haystacks

Given the large contribution of CNVs towards neurodevelopmental disorders, a study of rare CNVs in multiple disease cohorts would be a natural starting place to identify genes shared among the disorders. In this issue of *Genome Medicine*, Hakonarson and colleagues [3] examined CNVs present in 7849 patients from five neurodevelopmental and neuropsychiatric disease cohorts, which included schizophrenia, bipolar disease, autism, ADHD, and depression, in the first large scale meta-analysis of CNVs across these diseases. Given that the genetic basis of depression and bipolar disorder have been elusive, the inclusion of cohorts of individuals with these two disorders is especially informative. The authors processed microarray data from the five cohorts and performed a gene-by-gene assessment for enrichment of CNVs in each cohort. Using this gene-based analysis, the authors identified two loci, *ZNF280A* and *DOCK8*, which were significantly enriched in affected individuals for at least two of the disease cohorts. Deletions in the zinc-finger protein *ZNF280A* were enriched in the ADHD and autism cohorts, whereas duplications that encompassed *DOCK8* and the adjacent gene *KANK1* were enriched in all five cohorts, which represents the first novel shared locus on chromosome 9p24.3 for these disorders. Neither *DOCK8* nor *KANK1* have been previously identified as candidate disease loci, but these genes have roles in intracellular signaling and neural growth/migration, respectively, that indicate a possible role for these genes in neurodevelopment. As this is the first study to identify 9p24.3 as a candidate disease locus, follow-up studies and functional experiments are important steps to delineate the roles of *DOCK8* and *KANK1* in specific disorders.

**Fig. 1:**
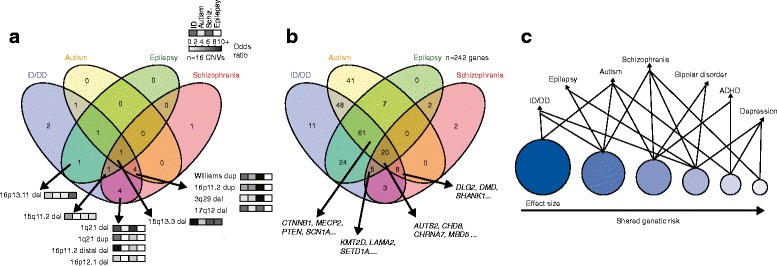
Shared genetic etiology in neurodevelopmental disorders. **a** Venn diagram showing associations for 16 rare pathogenic copy number variants (CNVs) identified in disease cohorts for four neurodevelopmental disorders: intellectual disability/developmental delay (ID/DD) [4], autism [5], schizophrenia [6], and epilepsy [7]. Odds ratios for each disorder were calculated for each CNV based on the study data, with an odds ratio > 2 used to assign a CNV to a particular neurodevelopmental disorder. The *grey scale bars* next to the highlighted CNVs represent odds ratios (in order from *left* to *right*) for intellectual disability, autism, schizophrenia, and epilepsy. **b** Venn diagram showing associations for 242 genes with at least one identified de novo loss-of-function variant or single-gene deletion found in neurodevelopmental disease cohorts [10]. **c** A model for the contributions of genetic variants with different effect sizes towards various neurodevelopmental disorders. Variants with larger effect sizes are likely to be primary causal variants, while variants with smaller effect sizes indicate modifiers that modulate the phenotype in concert with other variants

Identification of the same rare CNV across several disorders should come as no surprise, as several rare CNVs are already implicated in multiple neurodevelopmental disorders (Fig. 1a). For example, the 15q11.2 deletion has been identified in individuals with intellectual disability, schizophrenia, epilepsy, and ADHD, whereas the 16p11.2 deletion is a strong risk candidate for autism and also contributes to intellectual disability, epilepsy, and obesity at varying degrees of penetrance [4–7]. Mutations and structural variations that affect individual genes also appear to contribute towards multiple disorders (Fig. 1b). *DISC1* is a classic example of genetic pleiotropy, in which balanced translocations of the gene are associated with schizophrenia and bipolar disorder in multiple members of a large family [8]. In these instances, the varied clinical outcomes associated with the same causal variant are likely to be determined by disease-specific modifier variants in the genetic background.

## Primary causative variants or disease modifiers?

The novel duplication at 9p24.3 identified by Hakonarson and colleagues [3] could be a causative variant, but it is also possible that the duplication is a modifier for multiple neurodevelopmental outcomes (Fig. 1c). Modifier variants can be present in unaffected individuals but frequently co-occur with other known variants in affected individuals. These variants are usually just below the threshold for causation and have a lower effect size compared to causative variants. For example, deletions in *LRRC4C* were found to co-occur with other known pathogenic CNVs in individuals with autism, even though the variant by itself was not causal for the disease [9]. Combinations of variants of moderate or low effect size might be responsible for psychiatric phenotypes without known causative variants, such as bipolar disorder or depression, but variants of high effect size and potential causal nature are more likely to be associated with overt developmental disorders such as intellectual disability, autism, and schizophrenia. As the 9p24.3 duplication is enriched in individuals with both classes of neurodevelopmental disorders, the duplication could represent a novel modifier variant that interacts with causative variants in autism and schizophrenia and other low-effect variants in bipolar disorder and depression. Large-scale genomic studies such as that described by Hakonarson and colleagues [3], along with high-resolution phenotyping that evaluates multiple features of neurodevelopment, would be necessary to accurately determine whether a variant causes or modifies disease in specific contexts.

## Leveraging shared genetic features for more informed clinical management

The meta-analysis of five disorders presented by Hakonarson and colleagues [3] identified a novel causative or modifier locus associated with multiple disorders. Recognizing the pleiotropic effects of variants such as the 9p24.3 deletion, it is clear that there is a larger degree of shared heritability among neurodevelopmental diseases than that suggested by their diverse clinical features. Identification of shared genes would be useful to uncover common molecular pathways for these distinct disorders. Discovery of shared genes and pathways will enable researchers to more accurately diagnose individuals with multiple disorders, assess the risk of developing co-morbid features, and ultimately design therapeutic targets that can be used to treat multiple disorders in these individuals.

## Abbreviations

ADHD: Attention-deficit hyperactivity disorder
CNV: Copy number varian
SNV: Single-nucleotide variant
